# Ascites and Serum Interleukin-10 Levels as a Prognostic Tool for Ovarian Cancer Outcomes

**DOI:** 10.3390/cancers16162840

**Published:** 2024-08-14

**Authors:** Paul Adrien Guigue, Yoav Brezinov, Amber Yasmeen, Maroua Mbarik, Shannon Salvador, Susie Lau, Walter Henri Gotlieb, Melica Nourmoussavi Brodeur

**Affiliations:** 1 Lady Davis Institute for Medical Research at the Jewish General Hospital, McGill University, Montreal, QC H3T 1E2, Canada; 2 Department of Experimental Surgery, McGill University, Montreal, QC H3T 1E2, Canada; 3 Department of Obstetrics and Gynecology, McGill University, Montreal, QC H3T 1E2, Canada

**Keywords:** ovarian cancer, interleukin-10, ascites, serum

## Abstract

**Simple Summary:**

There are no reliable prognostic biomarkers for ovarian cancer. The immune tumor suppressive marker interleukin-10 has been shown to be elevated in cancer, specifically in ovarian cancer. This study aims to correlate interleukin-10 levels in the ascites and sera of ovarian cancer patients to correlate with cancer-related data and outcomes. Our findings suggest a prognostic role for interleukin-10 that may be related to its immunosuppressive function in the tumor microenvironment. Future studies are needed to validate these results. This study highlights a potential target for novel therapeutic approaches.

**Abstract:**

Interleukin-10 (IL-10) has been shown to be present at high levels in the ascites of ovarian cancer (OC) patients; however, little is known about its prognostic value. We sought to correlate IL-10 levels in ascites and sera of OC patients with clinicopathologic characteristics and oncologic outcomes. IL-10 levels and clinical data from biobanked ascites and serum samples of OC patients were evaluated. Receiver operating characteristic curves were used to quantify marker performance and identify IL-10-high and IL-10-low groups. Correlations between IL-10 levels and clinicopathologic data were performed. Survival outcomes were calculated, while the factors affecting them were also investigated. A total of 106 patients had ascites samples, of which 44 serum samples were also available. Mean ascites IL-10 levels were significantly higher in patients with serous histology compared to endometrioid histology (*p* = 0.024). Fold-change in ascites IL-10 during treatment positively correlated with clinical response, as determined by a change in serum cancer antigen (CA)-125 levels (*p* = 0.0126). Median progression-free survival (PFS) and overall survival (OS) were shorter in patients with high compared with low ascites IL-10 levels (PFS: 18 versus 60 months; *p* = 0.007, OS: 42 versus 85 months; *p* = 0.029). A significant positive correlation was seen between ascites and sera IL-10 levels (*p* = 0.019). In multivariable analyses, a high ascites IL-10 level was associated with a significantly worse prognosis (PFS hazard ratio (HR) = 1.93; *p* = 0.02). Patients with high ascites levels of IL-10 have worse outcomes, which are likely reflective of the immunosuppressive effect of IL-10. This highlights its potential role as an immunomodulator in the tumor microenvironment, leading to OC immune evasion.

## 1. Introduction

Epithelial ovarian cancer (OC) is the fifth leading cause of cancer-related deaths in the United States [1]. Patients often present with advanced-stage disease and undergo combination surgery and platinum-based chemotherapy in the first-line setting [2]. Recognizing that OC is a heterogenous disease entity, there is an active effort being made to shift towards more personalized treatment strategies [2]. Immune targeted therapies, including immune checkpoint inhibitors (ICIs), have shown dramatic success in multiple cancer types, including some gynecologic cancers such as endometrial [3,4] and cervical cancers [5]. To date, ICI trials in patients with OC have yielded disappointing response rates with no clear biomarker for response [6]. Despite its low specificity, serum cancer antigen (CA)-125 is still the most commonly used biochemical marker, utilized in OC studies to determine treatment response and correlated with survival [6,7]. The change in CA-125 levels rather than absolute values offers better predictive power; thus, this marker is actively used in the clinical setting to monitor response and recurrence [2,7]. 

Interleukins (ILs) are small signalling proteins that belong to a broad category of cytokines and are secreted by key immune cells that then mediate immunoregulatory pathways [8]. A unique feature of OC is the presence of excessive peritoneal cavity fluid, called ascites, that contains many cell subtypes, including cancer and immune cells, as well as many secreted cytokines [9]. Our group and others have described the presence of interleukin-10 (IL-10) in the ascites of patients with OC [9,10,11]. In the OC tumor microenvironment (TME), IL-10 was found to be secreted by mostly innate immune cells, such as monocytes, dendritic cells, macrophages and natural killer cells [8,12], and also adaptive immune cells such as CD4+ and CD8+ T cells, Th17 cells and B cells [8]. Increased expression of IL-10 can also occur in OC cells [8]. IL-10 has been shown in OC [13] to possess immunosuppressive properties, namely the impairment of antigen presentation [14,15], the promotion of cancer stemness with M2 polarization of macrophages and the Th2 cell response [8]. 

The modest clinical activity of immune targeted therapy observed in OC may thus be related to cytokine-induced immunosuppression, leading to an unresponsive TME. This highlights the need to better understand the factors that modulate the TME and impact response to treatment. Although some studies of cytokine markers suggest a possible association of IL-10 with certain clinicopathologic features in OC [16], the role of IL-10 in OC and its correlation to clinical outcomes are poorly understood. The aim of this study was to correlate IL-10 levels in ascites and sera of OC patients with clinicopathologic features, specifically stage, histology and CA-125 levels, as well as oncologic outcomes, including progression-free survival (PFS) and overall survival (OS).

## 2. Materials and Methods

### 2.1. Patients

A retrospective study was conducted, including all consecutive OC patients with available biobanked ascites samples between 2003 and 2023. All cases were included from a single centre, the Jewish General Hospital, in Montreal, Canada. The latter is an academic university-affiliated tertiary hospital. Samples from patients that had pre- and on-treatment ascites collected were included as well. Additionally, available serum samples of these OC cases were also included. 

### 2.2. Data Collection

All cases were reviewed by an experienced gynecologic pathologist. We collected the following data from electronic medical records: demographics (age, body mass index), clinicopathologic characteristics (tumor histology, stage, serum CA-125 level), treatment characteristics (type of surgery, surgical residual disease) and oncologic outcomes (follow-up CA-125 levels, disease recurrence, death). We used the American Society of Anesthesiologists (ASA) score as a proxy of performance status [17]. Surgical residual disease was recorded from the surgical reports. Disease progression was defined by either an increase in serum CA-125 ≥ 2-fold (the nadir value) on two occasions [18] or radiologic studies indicating the progression or appearance of new lesions. PFS and OS were defined by the time between sample collection and evidence of disease progression or death (any cause), respectively. We defined early-stage disease as stage I/II and advanced-stage disease as stage III/IV.

### 2.3. Sample Collection, Preparation and Storage

Following patient sample collection during hospital clinic or in-patient visits, ascites fluid and serum were rapid-centrifuged for 10 min in 1900× *g* for the removal of cellular debris and fibrin, and the cell-free supernatant was transferred to Eppendorf tubes, which were stored at −80 °C in the Gynecologic Tumor Biobank at the hospital’s affiliated research centre.

### 2.4. IL-10 Detection

IL-10 concentration in ascites and serum samples (with control standards) was quantified using enzyme-linked immunosorbent assay (ELISA) kits (BD Biosciences, Franklin Lakes, NJ, USA, cat #555157), according to the manufacturer’s protocol. All samples were analyzed in duplicates and the mean values were used for statistical analysis. 

### 2.5. Ethics Approval

This study was approved by the Institutional Review Board of the Jewish General Hospital (Protocol #2023-3591), in accordance with the Helsinki declaration. Informed consent was obtained from all patients to biobank specimens.

### 2.6. Statistical Analysis

We performed descriptive statistical analysis on the study cohort. The normality of the continuous data was assessed using the Shapiro–Wilk test. Data are presented as medians with interquartile range (IQR) for continuous variables, numbers with percentage for proportions and as medians with a 95% confidence interval (CI_95_) for survival data. Comparisons between continuous variables, when normality was not assumed, were performed using the Kruskal–Wallis or Wilcoxon Mann–Whitney tests. 

To determine the best cut-off at which IL-10 levels predict recurrence, receiver operating characteristic (ROC) curve analysis was performed. The maximal Kolmogorov–Smirnov metric was used to dichotomise the continuous variable, IL-10 levels, and distinguish IL-10-high and IL-10-low groups. We used a Spearman’s rank non-parametric test to study the correlation between the change in ascites IL-10 concentration and change in serum CA-125 concentration, before and during treatment. This test was also used to study the correlation between ascites and sera IL-10 levels. Spearman’s rank correlation coefficient, rho (r), was used to determine the strength of association between the two variables in a single value between −1 and +1. A positive correlation coefficient indicates a positive relationship between two variables, while a negative correlation coefficient indicates a negative relationship between two variables. A value of −1/+1 indicates a perfect relationship, while 0 indicates no relationship.

Survival estimates were plotted utilizing the Kaplan–Meier method. The log-rank test was utilized to quantify statistically these survival differences. Hazard ratios (HRs) with CI_95_ were obtained using the Cox proportional hazard model. Statistical significance was indicated by *p* < 0.05. Statistical analysis was performed using SPSS statistical software (IBM SPSS Statistics 29; Chicago, IL, USA), R version 4.1.2 (https://www.R-project.org/, accessed on 27 June 2024) and GraphPad Prism (version 8.0.0, GraphPad Software, San Diego, CA, USA).

## 3. Results

### 3.1. Ascites IL-10 Levels in OC Cohort

**Figure 1:**
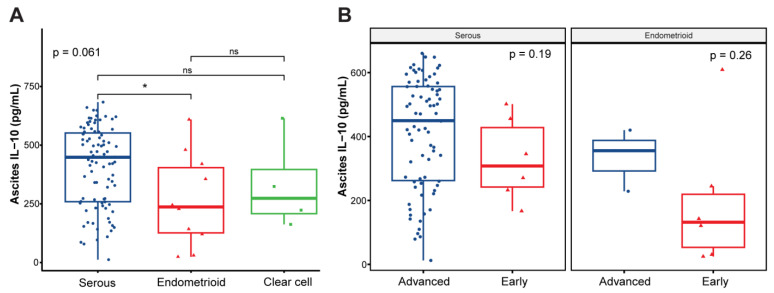
Ascites IL-10 levels in ovarian cancer patients. (**A**) IL-10 levels by histology. Statistical analysis was performed using Kruskal–Wallis and post hoc Mann–Whitney U tests. *N* = 91 (serous), *N* = 10 (endometrioid), *N* = 4 (clear cell). (**B**) IL-10 levels by stage and histology for serous and endometrioid subtypes. Statistical analysis was performed using Mann–Whitney U test. Early-stage disease = I/II and advanced-stage disease = III/IV. *p*-values are indicated on panel. *N* = 6 (serous early-stage), *N* = 78 (serous advanced-stage), *N* = 6 (endometrioid early-stage), N = 3 (endometrioid advanced-stage). Adenosquamous histology was not included in comparison analysis (*N* = 1). IL-10 levels were calculated using enzyme-linked immunosorbent assay. ns = not statistically significant; * *p* < 0.05.

Overall, there were 106 ascites samples included in our study; 98 samples were collected at the time of diagnosis and 8 were collected at the time of recurrence. Eighty-eight (83%) cases were high-grade serous, ten (9.4%) were endometroid, four (3.8%) were clear cell, three (2.8%) were low-grade serous and one (0.9%) was adenosquamous OC (Table 1). Most patients had advanced-stage disease (*N* = 92). The mean ascites IL-10 levels were statistically higher in the serous subtype of OCs compared to endometrioid histology (408.8 pg/mL versus 265.8 pg/mL; *p* = 0.024) but not compared to clear cell histology (331 pg/mL; *p* = 0.41) (Figure 1A). Furthermore, when stratified by stage, primary OCs showed a trend in higher IL-10 levels with more advanced disease (412.8 pg/mL in advanced stages versus 328.7 pg/mL in early stages for serous OCs; *p* = 0.19; Figure 1B). 

**Figure 2:**
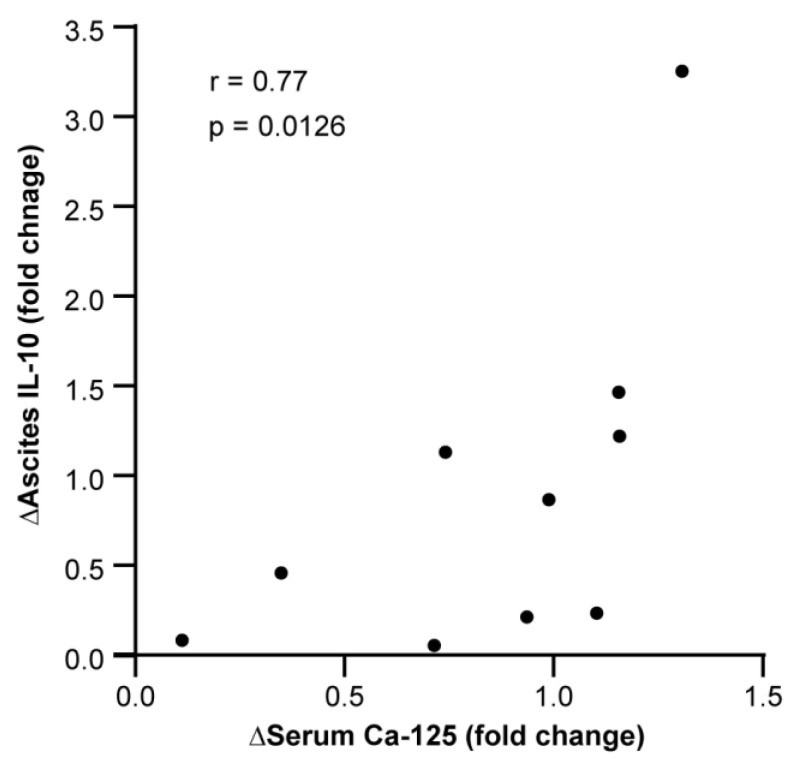
Correlation between change in ascites IL-10 levels and serum CA-125 levels. Fold change in CA-125 and IL-10 levels was determined by calculating on-treatment value/pre-treatment value (*N* = 10). Ascites and serum samples were collected on the same day. Statistical analysis was performed using Spearman’s correlation analysis with resulting rank correlation coefficient and two-tailed *p*-value indicated on the panel. Exact *p*-value indicated on figure. R-value = Spearman’s rank correlation coefficient.

We identified 10 cases that had a pre- and on-treatment ascites sampling and had corresponding serum CA-125 levels collected on the same days. As serum CA-125 level is currently the most used biomarker for therapy response [19], we compared the fold change in both markers. The change in ascites IL-10 concentrations correlated very closely with the change in serum CA-125 levels (r = 0.77, *p* = 0.0126; Figure 2). 

### 3.2. Survival Analysis

**Figure 3:**
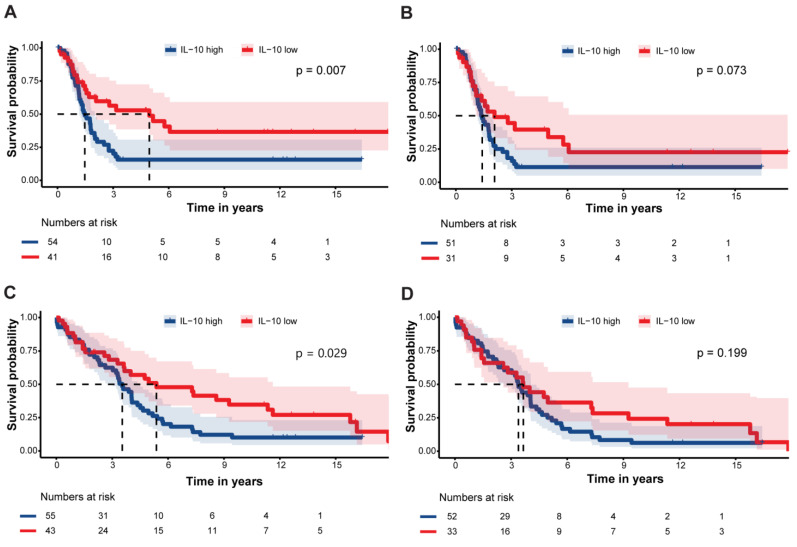
Survival outcomes of ovarian cancer patients by ascites IL-10 levels. Kaplan–Meier curves comparing (**A**) progression-free survival (PFS) in all patients, (**B**) PFS in patients with advanced-stage disease, (**C**) overall survival (OS) in all patients, and (**D**) OS in patients with advanced-stage disease. IL-10-high group refers to patients with concentrations ≥ 369 pg/mL, while IL-10-low group refers to patients with concentrations < 369 pg/mL. Survival was assessed in ovarian cancer patients with primary disease of all histologic types with available ascites samples (*N* = 98). *N* = 85 for advanced-stage disease patients. Three cases could not be analyzed in PFS calculations given that sample collection and progression had the same date (aggressive disease leading to imminent death). Survival was compared with log-rank test. Exact *p*-values indicated on figures. Dashed line refers to the time in years when 50% of the cohort is recurrence-free or alive.

The median follow-up time for the primary disease cohort was 40.6 months (IQR 17.2–75.7) during which there were 64 (65.3%) events of recurrence and 75 (76.5%) events of death. The ROC curve analysis best predicted the risk of recurrence at an ascites IL-10 value of ≥369 pg/mL (Figure S1A). The median PFS was significantly shorter among patients with high ascites IL-10 (≥369 pg/mL) compared to women with low ascites IL-10 (<369 pg/mL) levels (18 months, CI_95_ 14–24, versus 60 months, CI_95_ 20-not reached (NR), *p* = 0.007; Figure 3A). Amongst the patients with primary advanced-stage disease (*N* = 85), high IL-10 levels were associated with shorter median PFS (17 months, CI_95_ 14–22, versus 25 months, CI_95_ 13-NR, *p* = 0.073; Figure 3B). Similarly, the median OS was significantly shorter among patients with high ascites IL-10 compared to those with low ascites IL-10 levels (42 months, CI_95_ 36–54, versus 85 months, CI_95_ 43–144, *p* = 0.029; Figure 3C). OS was not statistically significantly different between groups in the advanced-stage patients (*p* = 0.20; Figure 3D).

Univariable analyses showed that high ascites IL-10 levels were a significant prognostic factor for short PFS and OS (HR = 2.05, CI_95_ 1.20–3.51, and HR = 1.69, CI_95_ 1.05–2.72, respectively), and remained statistically significant for short PFS on multivariable analyses (HR = 1.93, CI_95_ 1.11–3.38, *p* = 0.02; Table 2A).

### 3.3. Serum IL-10 Levels in OC Patients

**Figure 4:**
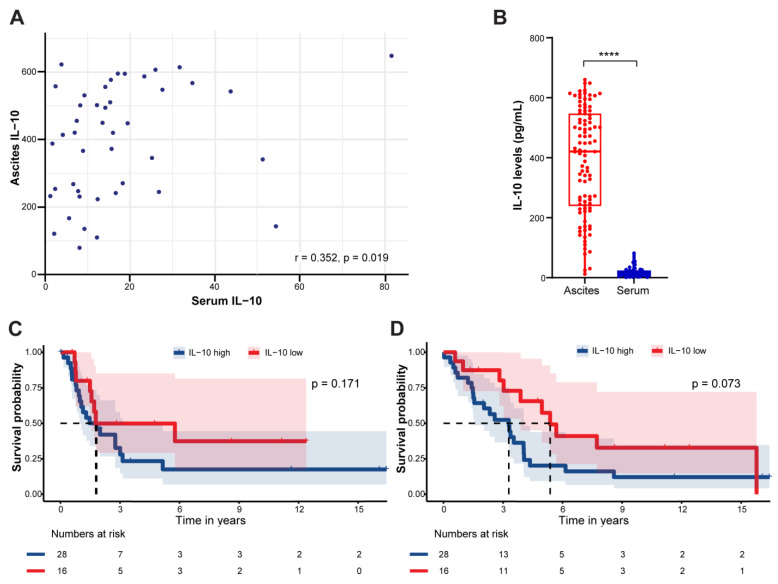
Outcomes of ovarian cancer patients by serum IL-10 levels. (**A**) Spearman’s correlation of ascites IL-10 levels and sera IL-10 levels. Statistical analysis was performed using Spearman’s correlation analysis with resulting rank correlation coefficient (R-value) and two-tailed *p*-value indicated on the panel. (**B**) IL-10 levels in ascites and sera of ovarian cancer patients with primary disease of all histologic types with available ascites samples (*N* = 98). Statistical analysis was performed using Mann–Whitney U test. Kaplan–Meier curves comparing (**C**) progression-free survival and (**D**) overall survival. IL-10-high group refers to patients with concentrations ≥ 9 pg/mL, while IL-10-low group refers to patients with concentrations of <9 pg/mL. Survival was assessed in 44 patients with ovarian cancer of all histologic types with available serum samples. Survival was compared with log-rank test. Exact *p*-values indicated on figures. Dashed line refers to the time in years when 50% of the cohort is recurrence-free or alive. **** *p* < 0.0001.

To determine if the findings in ascites could be observed in a less invasive liquid biopsy, we measured the IL-10 levels in the sera available for 44 of the 106 patients. Ascites IL-10 levels positively correlated with sera IL-10 levels (rho = 0.352, *p* = 0.019; Figure 4A). Overall, ascites IL-10 levels were significantly higher compared to sera IL-10 levels (*p* < 0.0001). The median follow-up time was 39.6 months (IQR 17.8–67.3), during which there were 28 (64%) events of recurrence and 33 (75%) events of death. The ROC curve analysis best predicted the risk of recurrence at a serum IL-10 value of ≥9 pg/mL (Figure S1B). The median OS was shorter among patients with high serum IL-10 (≥9 pg/mL) levels compared to patients with low serum IL-10 (<9 pg/mL) levels (40 months, CI_95_ 18–49, versus 65 months, CI_95_ 47–NR, *p* = 0.073; Figure 4B). Median PFS was similar between groups (22 months, CI_95_ 12–37, versus 22 months, CI_95_ 19-NR, *p* = 0.20; Figure 4C). Univariable analyses showed a trend that high serum IL-10 levels predicted shorter OS (HR = 1.96, CI_95_ 0.93–4.16), but this was not statistically significant, and similar results were found on multivariable analyses controlled for age, stage and histology (HR = 2.08, CI_95_ 0.93–4.63, *p* = 0.074; Table 2B).

## 4. Discussion

Overall, OC has poor survival rates with late initial presentation and frequent disease recurrences [20]. Targeted therapies available for OC patients are lacking compared to other cancer types. The disappointing immunotherapy trials in OC suggest an unresponsive TME, possibly due to immunosuppression. Uncovering the mechanisms of immune evasion is critical to improving immune targeted treatment strategies. In this study of chemotherapy naïve OC patients, significantly higher ascites IL-10 levels were seen in serous OCs compared to other histologies and furthermore in advanced-stage compared to early-stage serous OCs. Our results show that high ascites IL-10 levels are associated with shorter survival. Serum CA-125 is the most common biochemical prognostic marker used in OC, although it is not specifically a cancer biomarker [7]. Though it has not proven beneficial in the OC screening setting, it has demonstrated good predictive value in the therapy response and recurrence monitoring settings [2]. Patients in our cohort that had persistent high levels of IL-10 after starting treatment also had minimal change in their CA-125 levels, thus reflecting poor response to treatment. These data are hypothesis-generating and suggest that IL-10 may act as an immunomodulator based on its association with poor clinical outcomes, as suggested by others [21].

IL-10 plays a complex role in the tumor–immune cell intrinsic interactions and has been shown to be involved in the development and progression of several cancers, such as OC [8,11,14,22]. A previous smaller study found higher levels of IL-10 in OC compared to benign ovarian tumor or normal tissue [9]. Furthermore, studies evaluating serum cytokines, including IL-10, in OC patients showed that higher IL-10 levels were found in serous type of OCs [16,23] and in more advanced-stage disease [23]. Another group showed that ascites IL-10 levels were at least twice as high in advanced-stage disease compared to early-stage [9,11]. The prognostic impact of high IL-10 levels appears to be consistent across tumor type. Similar to our study, Reinartz et al. found that high ascites IL-10 levels in OC correlated with recurrence [24]. One study on Hodgkin’s disease also associated high IL-10 levels with poor survival [25]. Future work is necessary to validate our results in a larger cohort and determine whether the association of high IL-10 levels to poor clinical outcomes is due to an immunosuppressive effect on the TME.

The failure of major ICI trials to demonstrate efficacy in OC have alluded to the potential unique function of the ascites TME that contributes to immune escape mechanisms [26]. IL-10 appears to exhibit pro-tumorigenic properties in the TME. Preclinical studies have found that ascites IL-10 promotes tumor cell migration [27], and it is possible that IL-10 plays a major role in the immunosuppressed ascitic TME of OC [28,29,30]. Based upon our study and others, this would suggest that the IL-10 pathway could represent a therapeutic target. There is some evidence that IL-10 blockade, coupled with standard ICIs, has synergistic effects in various cancer models including OC [28,29,30]; hence, targeting the IL-10 pathway combined with ICIs may represent a potential treatment strategy in OC. Further research and mechanistic studies are needed to clarify the role of IL-10 as a an immunomodulator in the TME associated with poor outcomes and as a putative therapeutic target in OC.

Our study has some limitations inherent to its retrospective nature, including the inclusion of only one academic tertiary centre which limits external validity and the fact that serum samples were available for 44 of the 106 patients in this cohort. However, we have included all consecutive cases from our institution for which ascites and/or serum biospecimens were available. During the study period, clinical care has changed, including the use of robotic surgery and poly (adenosine diphosphate-ribose) polymerase inhibitors, which may impact clinical outcomes. Nevertheless, our OC cohort had a comprehensive correlation of ascites and serum IL-10 levels with oncologic outcomes. 

## 5. Conclusions

In conclusion, OC patients with high IL-10 levels in ascites have worse clinical outcomes. It is associated with serous histology, more advanced disease and shorter survival. Our data suggest that changes in ascites IL-10 levels reflect treatment response, as evidenced by comparing changes in serum CA-125 levels during treatment. Larger-scale cohort studies with long-term follow-up are required to confirm these results. Ascites IL-10 levels correlate closely to serum IL-10 levels, which may provide a non-invasive alternative that should be validating in a larger cohort of serum samples. It is uncertain whether IL-10 levels reflect the immunosuppressive state of the TME and would require further validation and mechanistic studies to confirm its potential immunomodulatory role.

## Figures and Tables

**Table 1:** Patient characteristics and demographics (*N* = 106).

Characteristics	*N* (%), Median (IQR)
Age (years)	63 (55–73)
Body mass index (kg/m^2^)	24 (21–28)
ASA ^a^	
1	5 (6.4)
2	45 (58)
3	26 (33)
4	2 (2.6)
Initial stage	
I	8 (7.5)
II	6 (5.7)
III	83 (78)
IV	9 (8.5)
Histology	
High-grade serous	88 (83)
Endometrioid	10 (9.4)
Clear cell	4 (3.8)
Low-grade serous	3 (2.8)
Adenosquamous	1 (0.9)
Surgery ^b^	
Primary cytoreduction	51 (50)
Interval cytoreduction	35 (34)
Secondary cytoreduction	1 (1.0)
No surgery	15 (15)
Residual surgical disease ^c,d^	
Optimal	76 (72)
Suboptimal	14 (13)

ASA = American Society of Anesthesiologist; IQR = interquartile range. ^a^ 28 patients did not have available ASA. ^b^ 4 cases did not have information on type of surgery performed. ^c^ 15 patients did not receive surgery. ^d^ Residual disease status missing for 1 case.

**Table 2:** Multivariable analyses for progression-free and overall survival. (**A**) Ascites samples only, *N* = 95 ^a^. (**B**) Serum samples only, *N* = 44.

**(A)**				
**Characteristic**	**Progression-Free Survival**		**Overall Survival**	
	HR (CI_95_)	*p*-value	HR (CI_95_)	*p*-value
Age	1.02 (1.00–1.05)	0.10	1.04 (1.02–1.06)	<0.001
Histology subtype				
Serous	Ref.		Ref.	
Endometrioid	0.25 (0.06–1.08)	0.063	0.51 (0.18–1.46)	0.21
Clear cell	2.59 (0.57–11.7)	0.22	3.01 (0.83–10.9)	0.092
Stage ^b^	2.82 (1.53–5.17)	<0.001	3.31 (1.85–5.93)	<0.001
Ascites IL-10 levels ^c^				
Low	Ref.		Ref.	
High	1.93 (1.11–3.38)	0.02	1.61 (0.99–2.63)	0.057
**(B)**				
Characteristic	Progression-Free Survival		Overall Survival	
	HR (CI_95_)	*p*-value	HR (CI_95_)	*p*-value
Age	1.01 (0.97–1.06)	0.60	1.04 (1.00–1.08)	0.084
Histology subtype ^d^				
Serous	Ref.		Ref.	
Endometrioid	0.80 (0.16–3.96)	0.78	0.67 (0.14–3.20)	0.62
Stage ^e^	2.82 (1.39–5.72)	0.004	2.41 (1.26–4.61)	0.008
Serum IL-10 levels ^f^				
Low	Ref.		Ref.	
High	1.55 (0.65–3.70)	0.32	2.08 (0.93–4.63)	0.074

^a^ Three cases could not be analyzed in PFS calculations, given that sample collection and progression had the same date (aggressive disease leading to imminent death). ^b^ Linear model used for ordinal variables. ^c^ Low defined as <369 pg/mL and high defined as ≥369 pg/mL. HR—hazard ratio; CI_95_—95% confidence interval. ^d^ Only 1 clear cell histology case with serum IL-10 samples was available; therefore, this subtype was excluded from analysis. ^e^ Linear model used for ordinal variables. ^f^ Low defined as <9 pg/mL and high defined as ≥9 pg/mL. HR—hazard ratio; CI_95_—95% confidence interval.

## Data Availability

The data supporting the findings of this study are available upon request from the corresponding authors.

## Supplementary Materials

The following supporting information can be downloaded at: https://www.mdpi.com/article/10.3390/cancers16162840/s1, Figure S1. Receiver operating characteristic curves.

## References

[1] Siegel R.L., Miller K.D., Wagle N.S., Jemal A. (2023). Cancer statistics, 2023. CA Cancer J. Clin..

[2] Lheureux S., Gourley C., Vergote I., Oza A.M. (2019). Epithelial ovarian cancer. Lancet.

[3] O’Malley D.M., Bariani G.M., Cassier P.A., Marabelle A., Hansen A.R., De Jesus Acosta A., Miller W.H., Safra T., Italiano A., Mileshkin L. (2022). Pembrolizumab in Patients with Microsatellite Instability-High Advanced Endometrial Cancer: Results From the KEYNOTE-158 Study. J. Clin. Oncol..

[4] Makker V., Colombo N., Casado Herráez A., Santin A.D., Colomba E., Miller D.S., Fujiwara K., Pignata S., Baron-Hay S., Ray-Coquard I. (2022). Lenvatinib plus Pembrolizumab for Advanced Endometrial Cancer. N. Engl. J. Med..

[5] Colombo N., Dubot C., Lorusso D., Caceres M.V., Hasegawa K., Shapira-Frommer R., Tewari K.S., Salman P., Hoyos Usta E., Yañez E. (2021). Pembrolizumab for Persistent, Recurrent, or Metastatic Cervical Cancer. N. Engl. J. Med..

[6] Matulonis U.A., Shapira-Frommer R., Santin A.D., Lisyanskaya A.S., Pignata S., Vergote I., Raspagliesi F., Sonke G.S., Birrer M., Provencher D.M. (2019). Antitumor activity and safety of pembrolizumab in patients with advanced recurrent ovarian cancer: Results from the phase II KEYNOTE-100 study. Ann. Oncol..

[7] Riedinger J.M., Bonnetain F., Basuyau J.P., Eche N., Larbre H., Dalifard I., Wafflart J., Ricolleau G., Pichon M.F. (2007). Change in CA 125 levels after the first cycle of induction chemotherapy is an independent predictor of epithelial ovarian tumour outcome. Ann. Oncol..

[8] Batchu R.B., Gruzdyn O.V., Kolli B.K., Dachepalli R., Umar P.S., Rai S.K., Singh N., Tavva P.S., Weaver D.W., Gruber S.A. (2021). IL-10 Signaling in the Tumor Microenvironment of Ovarian Cancer. Adv. Exp. Med. Biol..

[9] Zhou J., Ye F., Chen H., Lv W., Gan N. (2007). The expression of interleukin-10 in patients with primary ovarian epithelial carcinoma and in ovarian carcinoma cell lines. J. Int. Med. Res..

[10] Gotlieb W.H., Abrams J.S., Watson J.M., Velu T.J., Berek J.S., Martínez-Maza O. (1992). Presence of interleukin 10 (IL-10) in the ascites of patients with ovarian and other intra-abdominal cancers. Cytokine.

[11] Lane D., Matte I., Garde-Granger P., Bessette P., Piché A. (2018). Ascites IL-10 Promotes Ovarian Cancer Cell Migration. Cancer Microenviron..

[12] Wu L., Deng Z., Peng Y., Han L., Liu J., Wang L., Li B., Zhao J., Jiao S., Wei H. (2017). Ascites-derived IL-6 and IL-10 synergistically expand CD14. Oncotarget.

[13] Johnson R.L., Cummings M., Thangavelu A., Theophilou G., de Jong D., Orsi N.M. (2021). Barriers to Immunotherapy in Ovarian Cancer: Metabolic, Genomic, and Immune Perturbations in the Tumour Microenvironment. Cancers.

[14] Sato T., Terai M., Tamura Y., Alexeev V., Mastrangelo M.J., Selvan S.R. (2011). Interleukin 10 in the tumor microenvironment: A target for anticancer immunotherapy. Immunol. Res..

[15] Siminzar P., Tohidkia M.R., Eppard E., Vahidfar N., Tarighatnia A., Aghanejad A. (2023). Recent Trends in Diagnostic Biomarkers of Tumor Microenvironment. Mol. Imaging Biol..

[16] Mustea A., Könsgen D., Braicu E.I., Pirvulescu C., Sun P., Sofroni D., Lichtenegger W., Sehouli J. (2006). Expression of IL-10 in patients with ovarian carcinoma. Anticancer Res..

[17] Young J., Badgery-Parker T., Dobbins T., Jorgensen M., Gibbs P., Faragher I., Jones I., Currow D. (2015). Comparison of ECOG/WHO performance status and ASA score as a measure of functional status. J. Pain Symptom Manag..

[18] Rustin G.J., Nelstrop A.E., Tuxen M.K., Lambert H.E. (1996). Defining progression of ovarian carcinoma during follow-up according to CA 125: A North Thames Ovary Group Study. Ann. Oncol..

[19] Mogensen O. (1992). Prognostic value of CA 125 in advanced ovarian cancer. Gynecol. Oncol..

[20] Le Page C., Rahimi K., Köbel M., Tonin P.N., Meunier L., Portelance L., Bernard M., Nelson B.H., Bernardini M.Q., Bartlett J.M. (2018). Characteristics and outcome of the COEUR Canadian validation cohort for ovarian cancer biomarkers. BMC Cancer.

[21] Antoneeva I.I., Abakumova T.V., Dolgova D.R., Gening T.P., Pirmamedova S.S., Myasnikova D.F., Gening S.O. (2017). Cytokine Status of Serum in Ovarian Cancer Patients with Different Tumor Neoadjuvant Chemotherapy Response. Anticancer Agents Med. Chem..

[22] Huang Y., Zou K., Jiang H., Li Z. (2024). The complex role of IL-10 in malignant ascites: A review. Cancer Immunol. Immunother..

[23] Lambeck A.J., Crijns A.P., Leffers N., Sluiter W.J., ten Hoor K.A., Braid M., van der Zee A.G., Daemen T., Nijman H.W., Kast W.M. (2007). Serum cytokine profiling as a diagnostic and prognostic tool in ovarian cancer: A potential role for interleukin 7. Clin. Cancer Res..

[24] Reinartz S., Finkernagel F., Adhikary T., Rohnalter V., Schumann T., Schober Y., Nockher W.A., Nist A., Stiewe T., Jansen J.M. (2016). A transcriptome-based global map of signaling pathways in the ovarian cancer microenvironment associated with clinical outcome. Genome Biol..

[25] Sarris A.H., Kliche K.O., Pethambaram P., Preti A., Tucker S., Jackow C., Messina O., Pugh W., Hagemeister F.B., McLaughlin P. (1999). Interleukin-10 levels are often elevated in serum of adults with Hodgkin’s disease and are associated with inferior failure-free survival. Ann. Oncol..

[26] Ning F., Cole C.B., Annunziata C.M. (2020). Driving Immune Responses in the Ovarian Tumor Microenvironment. Front. Oncol..

[27] Gupta N., Liu J.R., Patel B., Solomon D.E., Vaidya B., Gupta V. (2016). Microfluidics-based 3D cell culture models: Utility in novel drug discovery and delivery research. Bioeng. Transl. Med..

[28] Lamichhane P., Karyampudi L., Shreeder B., Krempski J., Bahr D., Daum J., Kalli K.R., Goode E.L., Block M.S., Cannon M.J. (2017). IL10 Release upon PD-1 Blockade Sustains Immunosuppression in Ovarian Cancer. Cancer Res..

[29] Shen L., Li J., Liu Q., Song W., Zhang X., Tiruthani K., Hu H., Das M., Goodwin T.J., Liu R. (2018). Local Blockade of Interleukin 10 and C-X-C Motif Chemokine Ligand 12 with Nano-Delivery Promotes Antitumor Response in Murine Cancers. ACS Nano.

[30] Chen S., Wang X., Wu X., Wei M.Q., Zhang B., Liu X., Wang Y. (2014). IL-10 signalling blockade at the time of immunization inhibits Human papillomavirus 16 E7 transformed TC-1 tumour cells growth in mice. Cell Immunol..

