# Supplementary material for: Ascites and Serum Interleukin-10 Levels as a Prognostic Tool for Ovarian Cancer Outcomes
Source: Cancers (Basel). 2024 Aug 14;16(16):2840. doi: 10.3390/cancers16162840 (PMC11352926; doi:10.3390/cancers16162840)
Supplement: Supplementary file 1 [file cancers-16-02840-s001.zip › cancers-3110539-supplementary.pdf]

Supplemental Information

Ascites and serum interleukin-10 levels as a prognostic tool for ovarian cancer outcomes

Guigue PA et al

Supplemental Figures : Figure S1

SUPPLEMENTAL FIGURE

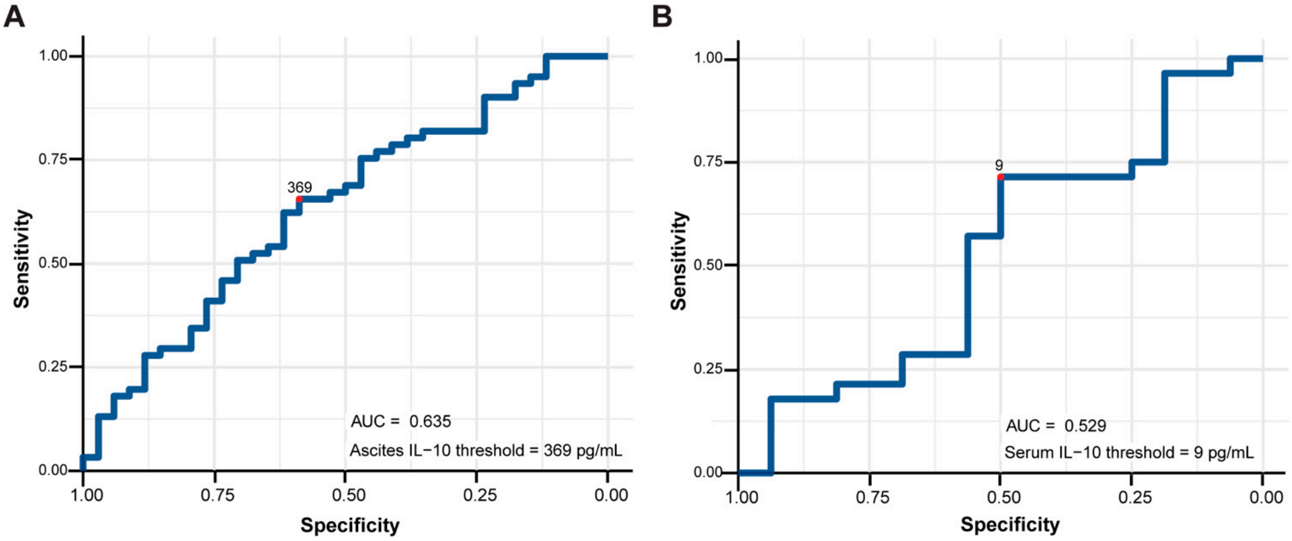

Figure S1. Receiver operating characteristic curves.

ROC curves for recurrence prediction of primary disease in (A) ascites (Area under the curve (AUC) = 0.635) and (B) sera (AUC = 0.529). Cut-off calculation based on maximum Kolmogorov-Smirnov. N = 95 for ascites cohort and N = 44 for sera cohort.
